# Research into acetone removal from air by biofiltration using a biofilter with straight structure plates

**DOI:** 10.1080/13102818.2015.1006413

**Published:** 2015-02-03

**Authors:** Pranas Baltrėnas, Alvydas Zagorskis, Antonas Misevičius

**Affiliations:** ^a^ Faculty of Environmental Engineering, Research Institute of Environment Protection, Vilnius Gediminas Technical University, Vilnius, Lithuania

**Keywords:** biofiltration, acetone, biofilter, wood fibre

## Abstract

The biological air treatment method is based on the biological destruction of organic compounds using certain cultures of microorganisms. This method is simple and may be applied in many branches of industry. The main element of biological air treatment devices is a filter charge. Tests were carried out using a new-generation laboratory air purifier with a plate structure. This purifier is called biofilter. The biofilter has a special system for packing material humidification which does not require additional energy inputs. In order to extend the packing material's durability, it was composed of thermally treated birch fibre. Pollutant (acetone) biodegradation occurred on thermally treated wood fibre in this research. According to the performed tests and the received results, the process of biodestruction was highly efficient. When acetone was passed through biofilter's packing material at 0.08 m s^−1^ rate, the efficiency of the biofiltration process was from 70% up to 90%. The species of bacteria capable of removing acetone vapour from the air, i.e. *Bacillus* (*B. cereus*, *B. subtilis*), *Pseudomonas* (*P. aeruginosa*, *P. putida*), *Stapylococcus* (*S. aureus*) and *Rhodococcus* sp., was identified in this study during the process of biofiltration. Their amount in the biological packing material changed from 1.6 × 10^7^ to 3.7 × 10^11^ CFU g^−1^.

## Introduction

Atmospheric emissions of volatile organic and inorganic compounds (acetone, xylene, ammonia, etc.) are relatively smaller than those of gaseous pollutants, such as carbon monoxide, carbon dioxide, nitrogen oxides or sulphur dioxide. Volatile organic compounds (VOCs), however, have greater influence on human beings and on the natural environment.[1–3] The release of VOCs into the environment from industrial facilities, e.g. foundries, rubber production, pharmaceutical and chemical industry, paint production plants, increases air pollution and the likelihood of smog formation.[4] In order to minimize the release of these pollutants into the environment, it is necessary to apply the most efficient means possible. One of such means is the application of air purification techniques. The optimum cleaning method is selected taking into account the suitability, efficiency and cost-effectiveness of the purification technique.

Considering the above-mentioned criteria (suitability, efficiency and cost-effectiveness); currently, the most attractive cleaning method is the treatment of volatile organic and inorganic compounds with biofilters. VOC treatment using a biofilter is based on the biofiltration technique. Biofiltration is a method for degradation of VOCs using certain cultures of microorganisms. Some of those microorganisms (bacteria) are *Pseudomonas fluorescens* and *Alcaligenes xylosoxidans*.[5] The bacterium *Pseudomonas putida* has been used to remove VOCs from the air. A cleaning efficiency of 90% has been achieved during these tests.[6] *Alcaligenes*, *Acinetobacter*, *Burkholderia*, *Pseudomonas*, *Xanthobacter* and *Hyphomicrobium* bacteria are suitable for the removal of VOCs from the air. The amount of bacteria in a biological packing material should range between 10^8^ and 10^10^ CFU g^−1^.[7]

One population of microbes suffices to degrade the VOCs.[8]

Microorganism's adaptation to a biological packing material may take from several days to several weeks.[9,10] Typically, the amount of bacteria in a biological packing material can range between 10^6^ and 10^10^ CFU g^−1^, while that of micromycetes between 10^3^ and 10^6^ CFU g^−1^.[11] The bacteria *Corynebacterium* and *Rhodococcus* are also suitable for VOC degradation.[12]

Experimental tests performed by Chan and Chang [13] with a VOC (acetone) at different pollutant concentrations (from 0.12 to 0.71 g m^−3^) show a maximum pollutant removal capacity of 95%.

Zhang and Pierce [14] have also used the bacterium *Rhodococcus* to degrade VOCs and the results of their research have shown a treatment efficiency of about 90%. Italian and Tunisian scientists also used the bacterium *Rhodococcus* for VOC removal. They also claimed that this bacterium is capable of treating VOCs and that treatment efficiency can range between 81% and 100%.[15]

In order for microorganisms to develop and remove VOCs from a polluted airflow, it is necessary to ensure favourable conditions for their growth and spreading on a biological packing material.

The physical factors that mostly have an influence on the growth and propagation of microorganisms are humidity and temperature.[16,17] Water is the most important medium in which the metabolism of organisms takes place; furthermore, all of the chemical reactions that occur in living microorganisms require water.

Since the packing material's humidity changes during VOC removal from the air, it is necessary to ensure the control of the humidification system in the biofilter. While flowing through the packing material, the air becomes saturated with vapour, eliminates humidity and reduces the moisture content of the packing material. However, at the same time, the process of biodegradation converts organic compounds into carbon dioxide (CO_2_) and water (H_2_O), thus partially restoring the humidity content. Decomposition of 1 kg of hydrocarbon produces 1.5 kg of water. Generally, this amount of water is insufficient for packing material humidification and, therefore, the packing material must be humidified additionally. To ensure an efficient process of pollutant biodegradation, the packing material's humidity has to reach 40%–70%.[18–20] To achieve an efficient performance of the biofilter, the humidity of the packing material has to reach 55%, while its porosity has to be 80%. The humidity of the packing material depends on its type and the humidification system used in the biofilter. In addition, the efficiency of the biofiltration process is highly influenced by the air temperature in the biofilter, as it determines the microorganism's development and activity. The air temperature in biofilters has to be distributed evenly within the volume of the biological packing material. In some biofilters this is achieved by installing air supply ducts across the entire area of the biological packing material, which requires greater financial inputs.[21]

Another important factor, necessary for efficient pollutant degradation, is the time of contact between the biological packing material and the pollutant. The longer the time of contact, the more efficient the biodestruction process.[22] The contact time depends on the thickness and porosity of the biological packing material. When the packing material in filters is thicker, but has lower porosity, the contact time is longer, but the aerodynamic resistance is higher.

An equally important factor is the pH of the medium. The transport mechanisms, reactions and growth rates of microorganism cells and also the destruction of some substances and the synthesis of others into new compounds depend on the acidity of the medium, which is defined by the pH measure. Most microorganisms tolerate ±1 to 2 pH deviation from the optimum value.[23] When the reaction of the medium changes, the activity of heterotrophic enzymes also changes. Neutral or weakly alkaline or acidic media with hydrogen ion concentration pH ranging between 6 and 8 are used for biological air treatment.[24] Most media, used to destroy volatile organic compounds, have a neutral concentration of hydrogen ions (pH = 7).

The aim of the research was to analyse the biofiltration process' efficiency by supplying acetone vapour with different concentrations to the biofilter. We applied a biofilter with plates of straight internal structure.

## Materials and methods

**Figure 1.**
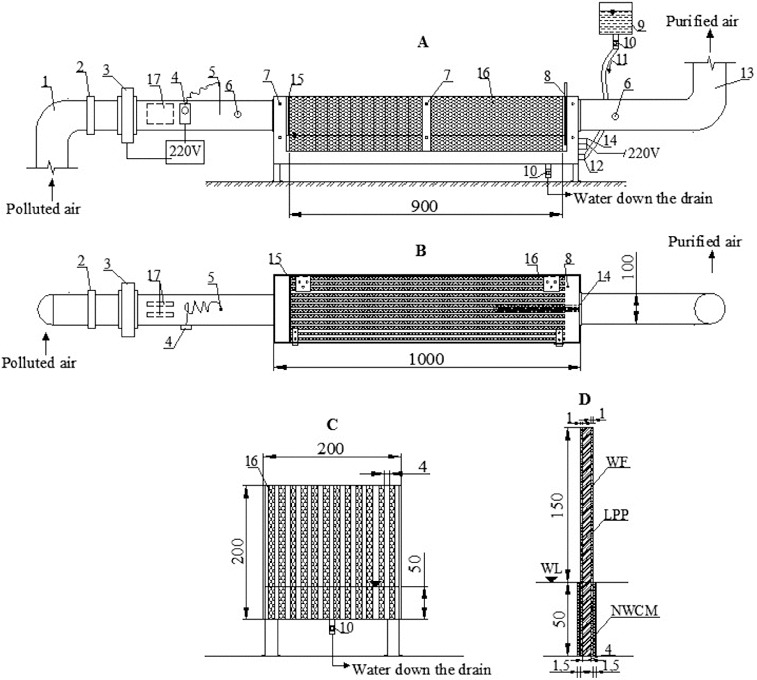
Chart of an air cleaning biofilter having a straight plate internal structure and capillary system for packing material humidification: side-view (a), view from above (b), view of biofilter cartridge (c), composition of the plate (d).

Note: 1 – polluted air duct; 2 – valve; 3 – ventilator; 4 – a thermal regulator; 5 – sensor; 6 – sampling holes; 7 – fixation elements; 8 – temperature sensor; 9 – tank; 10 – controllable valves; 11 – supply hose; 12 – controllable valves; 13 – clean air duct; 14 – biomedium heating element; 15 – perforated plate; 16 – biofilter cartridge; 17 – channel air heater; WL – water level, WB – wood fiber, NWCM – not-woven caulking material, LPP – linear polymer plate.

Experimental tests were carried out using a biological air purifier – biofilter. Figure 1 shows a chart of the biofilter with straight plates.

### Biofilter operation principle

Polluted air is supplied to the biofilter (Figure 1) via a polluted air duct (1) which is 100 mm in diameter. A ventilator (3) in the polluted air supply duct creates an airflow through the biofilter. The polluted air supply duct has a valve (2), which regulates the airflow velocity and, at the same time, the flow rate of the supplied air. Then, the polluted airflow enters a biofilter cartridge (16). The biofilter cartridge is packed with a packing material made of porous plates (Figure 1(d)). The airflow is evenly distributed by a perforated plate (15) over the entire volume of the packing material. The polluted air flows between the porous plates that are submersed in a liquid medium and arranged at 6 mm distance from each other. Having passed through the biofilter cartridge (16) with the packing material, a clean airflow enters a clean air duct (13), which is 100 mm in diameter, and is released into the environment. The cartridge is attached to the device by fixation elements (7). Sampling holes (6) are made in the polluted and clean air ducts. Airflow rate, temperature and pollutant concentrations supplied to and discharged from the biofilter are measured at these places. An excess of biomass is discharged from the biofilter via a biomass release valve (10). The required temperature of the supplied airflow is maintained by a channel air heater (17) with a thermal regulator (4) and a sensor (5). The biomedium temperature is maintained using a biomedium heating element (14) with a temperature sensor (8). A tank (9) with controllable valves (10, 12) and supply hose (11) is used to supply a solution saturated with biogenes to the biofilter.

The main element of the biofilter is the cartridge made of straight polymer plates on which packing material, i.e. wood fibre, is applied. The cartridge dimension is 900 ×  200 × 200 mm. Plates, which are arranged at 4 ± 0.2 mm distance from each other, produce the capillary effect of humidification. As a result of this effect, the water is capable of ascending through the pores of the wood fibre, when the space between the plates is small. During our research, the capillary system of packing material humidification was installed in the biofilter. Such humidification system has an advantage over other systems, in terms of significantly lower energy costs. Furthermore, it operates at a full capacity even when the power supply is discontinued.

In order to extend the durability of the birch fibre, it is necessary to undergo thermal processing. The birch fibre is obtained by thermal treatment of birch sawdust in a steam explosion reactor at a pressure of 3.2 × 10^6^ Pa and a temperature of 235 °C. Thus, changing the chemical structure of the wood prevents birch fibre from rotting in a humid medium, which results in an extension of the durability of the biofilter's packing material. The material was selected for its internal structure. The structure was determined by the method of electron microscopy. Scanning electron microscopy was performed using a field-emission scanning electron microscope JEOL ISM – 7600 F. Microscope enlargement was from × 25 to × 100,000. The electron acceleration voltage was from 0.1 to 30 kV. The image resolution was up to 5120 × 3840 pixels.

### Determining biomedium's pH and temperature

The required pH and temperature of the solution, saturated with biogenic elements, were maintained using KH_2_PO_4_ and K_2_HPO_4_ buffer solutions.[17] The values of pH and temperature were recorded on a daily basis. The porous plates of the biofilter cartridge were submersed into the solution, and saturated with biogenic elements (medium). The solution's composition used for the tests is presented in Table 1.

**Table 1.** Composition of aqueous biomedium.

Substance	Amount (g)
K_2_HPO_4_	1
KCl	0.5
MgSO_4_⋅7H_2_O	0.5
FeSO_4_⋅7H_2_O	0.1
NaNO_3_	0.9
Distilled water	1000

Such amounts of the used biogenic elements in the medium were selected after surveying data presented by other scientists during their research works.[22,25–29]

### Determining air humidity and temperature in the biofilter

Air temperature in the biofilter was measured by TESTO 400. This device can measure airflow velocity, rate, temperature, pressure and humidity. Airflow humidity and temperature were measured in three sections of the biofilter between the plates, i.e. at 150 mm from the airflow inlet (at 12 points), at 500 mm from the airflow inlet (12 points) and at 150 mm from the airflow outlet (12 points).

### Determining biological packing material's moisture content

The moisture of the biological packing material in the biofilter was determined by a moisture meter M0290. The parameter was measured at four points of each section every day of the experiment.

### Activating the biological packing material and determining the efficiency of the biodestruction process

During the conducted tests, an airflow polluted with acetone vapour was passed through the biofilter's packing material. The airflow rate between the wavy plates reached, on average, 0.08 m s^−1^. The same rate was also measured every day by the airflow meter Testo 400 with a thermocouple. The accuracy of thermocouple measurement is ±0.01 m s^−1^ when the airflow rate ranges between 0.01 and 2 m^3^ s^−1^.

The activation of the biological packing material lasted for 10 days. During the activation process, air polluted with acetone vapour was supplied to the biofilter. The initial pollutant concentration reached 0.256 g m^−3^. The pollutant was supplied to the device four times a day for 15 min, each time. During the next day, the concentration of the organic compound increased by 0.020 ± 0.005 g m^−3^ each time, by extending the duration of acetone vapour supply to 1 h. After the activation was completed, biofiltration efficiency tests were carried out for another five days, using air polluted with acetone vapour.

The efficiency of the pollutant decontamination was calculated by determining the pollutant concentration before entering the cleaning device and after leaving it.

### Determining the amount and composition of microorganisms in the biological packing material

A piece, weighing 1 g, was taken from each sample and placed into a flask containing 90 mL of 0.8% NaCl. In order to compare different samples with each other, calculations were made after the material in question was dried up to constant weight and the amount of microorganisms in 1 g of dry weight of the biofilter's material was calculated.

Micromycetes were grown on a medium agarized on beer mash. The cultures were incubated in Petri dishes for 5–7 days at a temperature of +28 °C.

Pure micromycete cultures were identified by classical methods, using descriptions by the following: for fungi – Ellis [30]; Pitt [31]; Watanabe [32]; Chaverri and Samuels [ 33 ]; Samson and Frisvad [34]; Domsch et al. [35]; Pečiulytė, Bridžiuvienė [36] and others.

Yeast was grown in the Sabouraud agar nutrient media with chloraphenicol (Liofilfem, Italy) and Rose Bengal CAF agar (Liofilchem, Italy). The culture was incubated in Petri dishes for 3–4 days at a temperature of +28°C.

Yeasts were identified using the identification systems Api 20 C AUX (bioMérieux, France).

We prepared nutrient agar (NA), selective agarized cetrimido (*Pseudomonas* (cetrimide)) agar and agarized *Bacillus cereus* media for the growth of bacteria from the analysed samples. The following bacterial suspensions were prepared: 1:10, 1:100, 1:1000, 1:10,000, 1:100,000, 1:1,000,000, 1:10,000,000, 1:100,000,000, 1:1,000,000,000 and 1:10,000,000,000. An amount of 0.1 mL of each suspension was poured onto the surface of the medium in Petri dishes and smoothed over with a spatula. The cultures were incubated for 2–4 days at a temperature of +28°C.

The grown bacteria were identified according to their morphological, biochemical and physical properties, and compared with the available reports. We used the descriptions of bacteria from Bergey's Manual of Systematic Bacteriology.[37–39]

### Analytical methods

The value of pH was determined according to the standard LST ISO 10523. A pH meter Mettler Toledo was used to determine the pH and temperature. The instrument's measurement range is from 0 to 14, and error is ±0.01. Airflow humidity and temperature were determined by TESTO 400. The range of airflow humidity measurement was from 0% to 100%, and the accuracy was 0.1%. The airflow temperature measurement range was from 200 to 800 °C. Packing material humidity was determined with a humidity meter (Extech Instruments, model MO290). The material humidity measurement ranges from 0% to 99.9% (error is ±0.1%). The pollutant's concentration was measured with the instrument MiniRae 2000 whose measurement limits range from 0 to 7.00 g m^−3^. The accuracy of measurement at pollutant's concentration from 0 to 0.100 g m^−3^ is 0.0001 g m^−3^, while at pollutant's concentration above 0.100 g m^−3^ is 0.001 g m^−3^. The method of washing (serial dilution method) was used to separate microorganisms and calculate their amount.

### Operating conditions

Studies were performed on the level of the acetone inlet load rate (ILR), the corresponding acetone removal efficiency (RE) and elimination capacity (EC). The definitions for these three parameters are set out as follows:

(1) $$\text{ILR}=\frac{Q\cdot C_{I}}{V}$$

(2) $$\text{RE}=\left(1-\frac{C_{0}}{C_{I}}\right)\cdot 100$$

(3) $$\text{EC}=\frac{Q\cdot \left(C_{I}-C_{0}\right)}{V}$$

where *C_I_* is the inlet acetone concentration in the biofilter (g m^−3^); *C*_0_ is the outlet acetone concentration in the biofilter (g m^−3^); ILR is the inlet loading rate (g m^3^ h^−1^; m s^−1^); *V* is the volume of the filter bed (m^3^) and *Q* is the gas flow rate (m^3^ h^−1^).

All of these parameters were studied in accordance with the operating conditions, as summarized in Table 2.

**Table 2.** Biofilter operating conditions.

Filtering medium	Wood fiber
Pollutant	Acetone
Cartridge dimensions	900 × 200 × 200 mm
Inlet concentration	0.025–0.997 g m^−3^
Airflow rate	3.4 m^3^ h^−1^
Residence time	11.2 s

## Results and discussion

The whole period of experiments (15 days) was divided into five stages (A, B, C, D and E). During Stage A, the biomedium was activated with acetone vapour by increasing the pollutant's concentration with 0.020 ± 0.005 g m^−3^ each day from Day 1 until Day 10 of the experiment. After Day 10 of the experiment, the efficiency of the biofiltration process was analysed (Stages B, C, D and E) (Table 3) by increasing the pollutant's concentration in the supplied air and maintaining a steady flow of the air that was supplied to the biofilter.

**Table 3.** Operating sections of the biofilter.

Section	Gas flow rate (m^3^ h^−1^)	Empty bed residence time EBRT (s)	Time (days)	Inlet concentration (g m^−3^)
A	3.4	11.2	1–10	0.025–0.277
B			10–12	0.296–0.310
C			12–13	0.451
D			13–14	0.634
E			14–15	0.997

**Figure 2.**
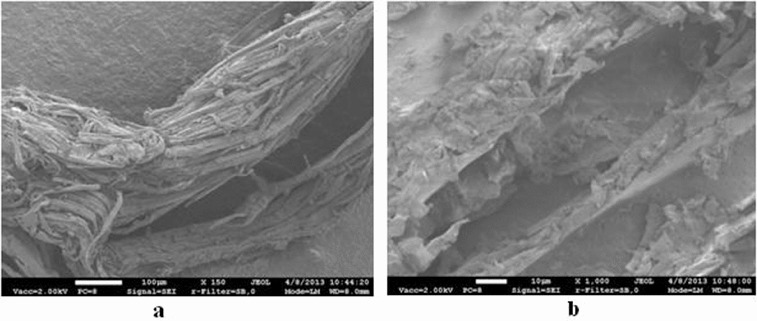
Structure of the birch fibre: 150 times magnification (a), 1000 times magnification (b).

The biological packing material is one of the main elements of the biofilter. Before undertaking any investigations, it is very important to analyse the structure of the packing material which determines the physical properties of the biomedium on which the efficiency of biofiltration process depends. The most important properties of the biomedium are porosity and capillarity. The greater the porosity of the material, the better the adsorption of pollutants from a polluted airflow.[40] When the porosity is higher, the capillarity is also more efficient. The better the parameters, the more efficient the pollutant biodegradation. Thermally treated birch fibre also has an uneven surface (Figure 2(a)), a porous structure (Figure 2(b)) and, at the same time, a bigger specific surface area.

As seen from the picture, it seems as if birch fibre is composed of many minor ‘straws’ arranged in parallel to each other, which are 15–30 µm thick. Wood fibre is necessary for the packing material in order for the microorganisms in the biomedium to assimilate organic carbon from it.

**Figure 3.**
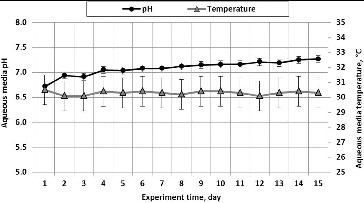
Medium pH and temperature during the time of passage of air polluted with acetone vapour through the biological packing material composed of wood fibre.

**Figure 4.**
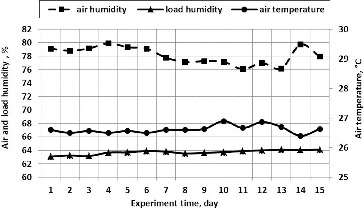
The humidity and temperature of the airflow during the cleaning and the humidity of the biological packing material.

**Figure 5.**
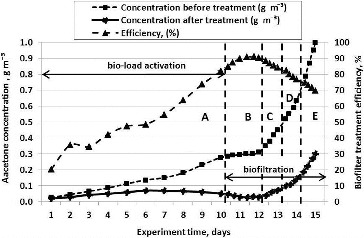
Relationships between the biofilter's air cleaning efficiency and concentration of the pollutant supplied to the biofilter when straight plates and packing material composed of wood fibre are used.

**Figure 6.**
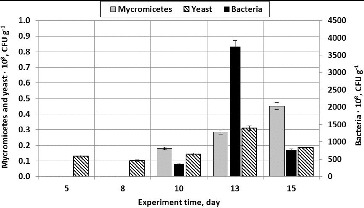
The amount of micromycetes, yeasts and bacteria (CFU g^−1^) when using straight plates and packing material composed of wood fibre (treated pollutant – acetone).

**Figure 7.**
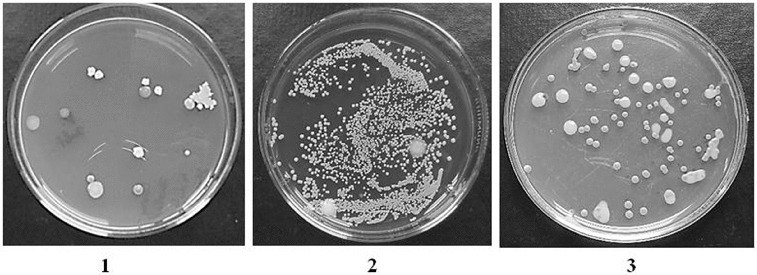
Bacteria composition by species.

Note: 1 – bacteria of the species *Bacillus subtilis*, 2 – bacteria of the species *Staphylococcus aureus*, 3 – bacteria of the genus *Rhodococcus* sp.

The Figures 3–7 show the results of experimental tests during which air polluted with acetone vapour was filtered through the biofilter having a straight internal structure.

The initial pH of the medium was 6.72 (Figure 3) and it depended on the chemical substances in the medium. During the experiment, the pH value of the medium increased and reached 7.27 on the experiment's Day 15. The temperature of the nutrient media in the biofilter was 30.2 °C. Therefore, taking into account the recommended pH limits for VOCs' degradation, the optimum pH level was ensured during this experiment. Many other scientists also maintain that the optimum pH for bacterial growth should reach 7.[41]

After comparing the results of our tests and the results of tests performed by American scientists who investigated a biofilter and removed VOCs from an airflow, it can be stated that we obtained similar results, which differ by a mere 4%.[42]

For the purpose of ensuring optimum conditions for the process of biofiltration, airflow humidity and temperature were measured (Figure 4).

Biological air purification efficiency greatly depends on a humidification system installed in biofilters.[43–45] In our research, a self-humidification system of the packing material was used in the biofilter which is based on the effect of capillary humidification of the packing material. The depth of porous plate soaking was 50 mm. The overall height of the plate was 200 mm. The porous structure of the plates and the size of the spaces (4 ± 0.2 mm) between the plates arranged next to each other produce the capillary effect of the packing material humidification. As a result of the effect of the packing material's capillary humidification, the solution (biomedium) spontaneously ascends and humidifies the wood fibre. Therefore, this self-humidification system does not use additional energy and ensures the appropriate humidification of the packing material even when the technological process is interrupted during repair work or when the power supply is discontinued for some other reasons. At the beginning of the experiment, the humidity of the biological packing material reached 63.1% and rose up to 63.7% on Day 4 and afterwards remained steady, representing, on the average, 63.8%. For ammonia removal, the Taiwanese scientist Chung [46] used a compost-packed biofilter. The results of the research showed that at 40%–46% humidity of the packing material, the air purification efficiency was high – 98%. It can be stated, therefore, that during our experiments, the humidity of the packing material was also sufficient. Since the recommended packing material humidity of 40%–70% was ensured, it had no adverse effect on the degradation of microorganisms in the biological packing material.

Temperature is the most important factor, responsible for the rate of the microorganisms' development and the intensity of biochemical reactions. Different groups of microorganisms are adapted for living at different temperatures. While analysing the biofilter's cleaning efficiency, in order to achieve a high cleaning efficiency, Taiwanese scientists Chang and Lu [27] recommend maintaining air humidity from 85% to 95% and temperature from 25 to 35 °C in the biofilter. According to many scientists, a change in the rate of pollutant degradation is not observed at a temperature of 20–30 °C.[47] However, where the temperature of the supplied airflow is higher than 40 °C, microorganisms may die, unless the microorganisms that are incubated in the biological packing material are thermophilic.[48] When the temperature of the discharged air increases or decreases, both the temperature of the supplied airflow and the fact that microorganisms emit heat during pollutant degradation can be important. Consequently, an increase or decrease in the amount of microorganisms in the biological packing material can result in changes in the air temperature.[49] The most efficient biodegradation of toluene in the polluted airflow was achieved at an airflow temperature of 30–35 °C.[50]

As seen from Figure 4, during our experiments, the airflow humidity in the biofilter between the straight plates varied in the range between 76% and 80%, and the temperature was between 26 and 27 °C.

The main factor responsible for the device's efficiency is its capability to remove pollutants (acetone) from a supplied airflow with the help of microorganisms.

Prior to operating biological air purifiers, the biological packing material inside them undergoes а biological activation. The packing material is activated, when the organic pollutants air is passed through it.[16] A packing material is considered to be biologically activated when it is covered by a thin layer (5–30 µm thick) of biofilm, which contains microorganisms.

In this case, the packing material was activated until experiment's Day 10. The packing material was activated by air polluted with acetone, which passed through it and gradually increased its concentration and recording the biofilter's cleaning efficiency.

The initial pollutant concentration was 0.0256 g m^−3^, while at the end of the experiment the concentration reached 0.997 g m^−3^ (Figure 5). As the figure shows, the biofilter's cleaning efficiency gradually increased until Day 11 of the experiment and approached the limit of 90.3%. On that day, the concentration of acetone vapour stood at 0.295 g m^−3^. Later, when the concentration of pollutant in the supplied air increased, the biofilter's cleaning efficiency decreased with 7% every day until Day 15 of the experiment. On Day 15 of the experiment, the efficiency of acetone vapour removal reached 69.8%. Air cleaning efficiency decreased due to the fact that the amount of bacteria in the biological packing material decreased from 3.7·× 10^11^ to 7.6·× 10^10^ CFU g^−1^. Since there was an excess of pollutants, microorganisms were incapable of degrading such amount thereof, and therefore, their death was observed according to the decrease of their number.

Empty bed residence time (EBRT) has a major impact on air cleaning efficiency.[51] Most experimental tests show that when the residence time increases, the VOCs' removal efficiency improves.[52,53] In order to achieve the longest possible residence time, it is recommended to increase the volume of the filtering layer. Also, the residence time depends on the level of biological decomposition.[49] During our tests, the residence time reached 11 s.

When acetone vapour concentrations were supplied to the biofilter in Stages B, C, D and E, the pollutant elimination capacity ranged between 33 and 87 g m^−3^ h^−1^. In Stage A, the biofilter's elimination capacity was from 0.6 to 28 g m^−3^ h^−1^.

At the beginning of the experiment, yeasts dominated and only one or two fungi colonies grew. However, after 10 days both the amount of fungi and the variety of their species increased. In addition to *Paecilomyces variotii*, which are distinguished by high-level sporulation, yeast fungi of the genera *Aureobasidium* and *Geotrichum* also developed well.

It has been determined that during volatile substance (acetone) filtration, the yeast *Rhodotorula mucilaginosa* dominated. The yeast amount ranged from 0.13·× 10^8^ CFU g^−1^ at the beginning of the experiment to 0.45·× 10^8^ CFU g^−1^ at its end. It has been determined during the analysis of the amount of bacteria on birch fibre that their amount grows from 0.16·× 10^8^ CFU g^−1^ on Day 5 to 0.83·× 10^8^ CFU g^−1^ on Day 13 (Figure 6).

Bacteria of the dominating genera and species were determined during the research. The largest amount of the determined bacteria belonged to the genera *Bacillus* (*B. cereus, B. subtilis*), *Pseudomonas* (*P. aeruginosa, P. putida)*, *Stapylococcus* (*S. aureus*) and *Rhodococcus* sp (Figure 7).

## Conclusions

When wood fibre was used for the biofilter's packing material, the optimum parameters ensuring an efficient work of microorganisms were maintained. Packing material's humidity reached 63.7% ± 1%, airflow temperature was 26.6 ± 2 °C, air humidity was 78.1% ± 5%, the medium pH was 7.1% ± 0.6% and the medium temperature was 30.3 ± 0.1°C.

The efficiency of removing acetone vapour from the air was between 70% and 90%. The highest air cleaning efficiency of 90.3% was achieved at 0.3 g m^−3^ concentration of pollutant supplied to the biofilter at 0.08 m s^−1^ rate.

When spontaneous microorganisms were used to achieve air cleaning efficiency, the bacteria *Bacillus* (*B. cereus, B. subtilis*)*, Pseudomonas* (*P. aeruginosa, P. putida), Stapylococcus* (*S. aureus*) and *Rhodococcus* sp. best adapted, developed and degraded acetone.
